# Metabolic Switching of Cultured Mesenchymal Stem Cells Creates Super Mitochondria in Rescuing Ischemic Neurons

**DOI:** 10.1007/s12017-022-08720-3

**Published:** 2022-07-20

**Authors:** Anna Gorsky, Molly Monsour, Hung Nguyen, Vanessa Castelli, Jea-Young Lee, Cesar V. Borlongan

**Affiliations:** 1 Center of Excellence for Aging and Brain Repair, Department of Neurosurgery and Brain Repair, University of South Florida Morsani College of Medicine, 12901 Bruce B Downs Blvd, Tampa, FL 33612 USA; 2 University of South Florida Morsani College of Medicine, 560 Channelside Dr, Tampa, FL 33602 USA

**Keywords:** Stroke, Mesenchymal stem cells, Mitochondria, Ischemic tolerance

## Abstract

Transfer of healthy mitochondria from mesenchymal stem cells (MSCs) to ischemic neurons represents a potent stroke therapeutic. MSCs were grown under ambient conditions (nMSCs) or a metabolic switching paradigm by alternating galactose and glucose in medium (sMSCs) and then assayed for oxygen consumption rates using the Seahorse technology. Subsequently, primary neurons were subjected to oxygen glucose deprivation (OGD) and then co-cultured with either nMSCs or sMSCs. Compared to nMSCs, sMSCs displayed higher basal energy production, larger spare respiratory capacity, greater ATP production, and decreased proton leak. Co-culture of OGD-exposed neurons with sMSCs conferred greater cell viability, enhanced cell metabolism, reduced mitochondrial reactive oxidative species mRNA, and elevated mitochondria ATP mRNA than those cultured with nMSCs. Metabolic switching produces “super” mitochondria that may underlie the therapeutic benefit of using sMSCs to treat ischemic cells.

**Supplementary Information:**

The online version contains supplementary material available at 10.1007/s12017-022-08720-3.

## Introduction

Stroke is the 5th leading cause of death and the most prevalent cause of disability in the United States (US). The few primary stroke treatments, including tissue-type plasminogen activator (tPA) or endovascular interventions, must be given in short-time windows and possess high risks (Kaesmacher et al., 2017; Lees et al., 2010). Probing ischemic cell death may offer insights on developing novel stroke treatments.

Mitochondrial damage stands as one of the primary promoters of secondary cell death (Wang & Youle, 2009). Recognizing that mitochondrial transfer between healthy cells and ischemic cells can attenuate stroke deficits (Aguer et al., 2011; Nguyen et al., 2019), we explored a potential mitochondrial-based therapy for ischemic stroke. Here, we found that MSCs grown under metabolic switching (sMSCs), with alternate medium supplementation of glucose and galactose in cell culture developed “super mitochondria” (sMito). These sMito likely played a major role in sMSCs superior amelioration of OGD deficits compared to OGD neurons treated with normal MSCs grown in ambient cell culture condition (nMSCs).

## Methods

The general experimental design consisted of two studies (Supplementary Materials). First, we cultured MSCs either under normal ambient cell culture condition (nMSCs) or under the metabolic switching paradigm (sMSCs). We assayed these two groups of MSCs for oxygen consumption rates to reveal potential differences in their mitochondrial respiration. Second, we exposed primary rat cortical cells to OGD and then co-cultured these neurons either with nMSCs or sMSCs. We then assessed the neurons for cell viability, metabolic activity, and mRNA levels of mitochondrial reactive oxygen species (mtROS) and mitochondrial ATP (mtATP). All investigators were blinded to the treatment conditions. Assignment of MSCs to either ambient or metabolic switching was conducted randomly (Supplementary Fig. 1). Treatment effects were measured with one-way ANOVA and Post hoc Bonferonni’s tests.

## Results

**Fig. 1:**
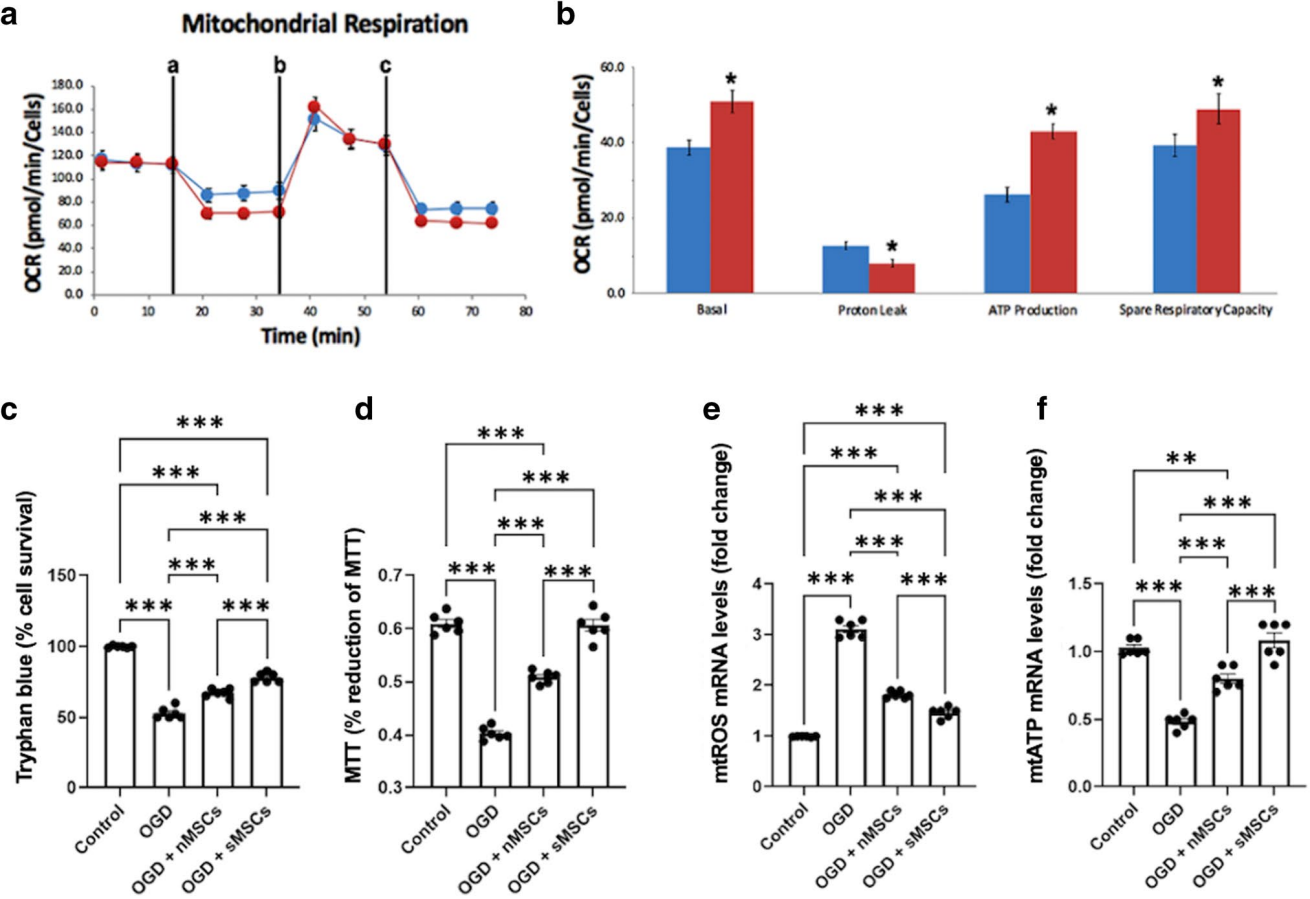
Mitochondrial respiration and enhanced functionality. **a** Line chart shows the oxygen consumption rate (OCR) changes in rate mode comparing MSCs grown under ambient cell culture condition (blue) and MSCs exposed to metabolic switching condition (red). **b** Bar chart corresponds to mitochondrial respiration changes in both groups of MSCs, which were analyzed with basal respiration, proton leak (a: oligomycin), ATP production (b: carbonyl cyanide-4 phenylhydrazone), and spare respiratory capacity (c: antimycin A & rotenone). Analyses included one-way ANOVA followed by Bonferonni’s pairwise tests. *n* = 6 biological replicates. **c** Trypan blue. ANOVA revealed significant treatment effects. Post hoc Bonferonni’s tests revealed significant differences in cell survival, with primary neurons exposed to OGD and co-cultured with sMSCs grown under metabolic switching (OGD + sMito) rescuing against OGD-induced cell death significantly better than nMSCs grown under ambient condition (OGD + nMito). **d** MTT. Similarly, ANOVA showed significant treatment effects with post hoc Bonferonni’s tests detecting significant differences in metabolic activity, again with primary neurons exposed to OGD and co-cultured with sMSCs grown under metabolic switching (OGD + sMito) preventing the OGD-induced metabolic impairment significantly better than nMSCs grown under ambient condition (OGD + nMito). **e** mtROS mRNA. ANOVA revealed significant treatment effects. Post hoc Bonferonni’s test showed significant differences in mtROS mRNA levels, with primary neurons exposed to OGD and co-cultured with sMSCs grown under metabolic switching (OGD + sMito) dampening the OGD-induced mtROS mRNA elevation significantly better than nMSCs grown under ambient condition (OGD + nMito). **f** ATP mRNA. Similarly, ANOVA shows significant treatment effects with post hoc Bonferonni’s tests detecting significant differences in ATP mRNA levels, again with primary neurons exposed to OGD and co-cultured with sMSCs grown under metabolic switching (OGD + sMito) preventing the OGD-induced ATP mRNA downregulation significantly better than nMSCs grown under ambient condition (OGD + nMito). *p’s < 0.05, **p’s < 0.01, ***p’s < 0.001

We first cultured MSCs in glucose-supplemented medium (normal MSCs) (nMSCs) or in a medium with metabolic switching from glucose and galactose every 3 days (switched MSCs) (sMSCs) to examine whether the MSCs would generate “super mitochondria” (Supplementary Fig. 2). Using the Agilent Seahorse XF Mito Stress Kit, we quantified the oxidative phosphorylation efficacy of the cells (Fig. 1a). There were significant treatment effects (*F*_3, 68_ = 455.80). Compared to the nMSCs, the sMSCs exhibited a significantly greater basal metabolic rate (*p* < 0.05), lower H+ leak (*p* < 0.05), greater ATP production (*p* < 0.05), and greater spare respiratory capacity (SRC) (*p* < 0.05) (Fig. 1b). These results demonstrate that the sMSCs’ super mitochondria (sMito) produced more energy and displayed OCR with coupled respiration or oxidative phosphorylation than nMSCs’ mitochondria (nMito).

With enhanced mitochondrial functionality in the sMSCs, we next deprived neurons of oxygen and glucose to develop an ischemic cell model. Neurons were treated with nMSCs or sMSCs. A control group of neurons was used with no OGD and no MSCs treatment. Cell viability was measured with Trypan blue (Fig. 1c). ANOVA revealed significant treatment effects (*F*_3, 20_ = 260.10). Compared to the control, OGD neurons (*p* < 0.001), OGD neurons with nMSCs (*p* < 0.001), and OGD neurons with sMSCs (*p* < 0.001) exhibited lower cell viability. sMSCs elevated cell viability compared to OGD neurons (*p* < 0.001) and compared to nMSCs (*p* < 0.001). Thus, sMSCs improved cell viability after OGD better than nMSCs, but not on par with control neurons.

As a measure of cell metabolism, we determined the neurons’ enzymatic reduction of tetrazolium salt 3-(4,5-dimethylthiazol)-2,5-diphenyltetrazolium bromide (MTT) (Fig. 1d). ANOVA showed significant treatment effects (*F*_3, 20_ = 175.40), with OGD neurons (*p* < 0.001) and nMSC-treated OGD neurons (*p* < 0.001) displaying lower metabolic activity compared to controls. OGD neurons (*p* < 0.001) and nMSC-treated OGD neurons (*p* < 0.001) showed lower metabolic activity compared to sMSC-treated OGD neurons. OGD neurons with nMSCs recovered metabolic activity somewhat (*p* < 0.001) but not to control levels (*p* < 0.001). OGD neurons treated with sMSCs increased the metabolic activity compared to OGD neurons (*p* < 0.001) and did not differ significantly from the control group. Overall, although nMSCs reversed ischemic effects, sMSCs provided an enhanced cell recovery.

Building on the successful demonstration of the potential use of sMSCs to reverse ischemic stroke damage, we investigated the downstream pathways of enhanced mitochondrial function. To determine how the exposure to MSCs with healthy mitochondria conferred enhanced cell recovery, we measured mtROS mRNA levels (Fig. 1e) and mtATP mRNA levels (Fig. 1f). ANOVA revealed significant treatment effects for mtROS mRNA levels (*F*_3, 20_ = 452.50) and mtATP mRNA levels (*F*_3, 20_ = 58.60). Compared to the control, OGD neurons (*p* < 0.001), OGD neurons with nMSCs (*p* < 0.001), and OGD neurons with sMSCs (*p* < 0.001) exerted greater levels of mtROS mRNA. Both nMSCs (*p* < 0.001) and sMSCs (*p* < 0.001) were able to reduce mtROS mRNA after OGD. sMSCs showed greater reduction than nMSCs (*p* < 0.001), but still differed from the control (*p* < 0.001). Thus, sMSCs were effectively better than nMSCs at reducing ROS damage after OGD, but still did not recover ROS to control levels. In a similar fashion, compared to the control, OGD neurons (*p* < 0.001) and OGD neurons with nMSCs (*p* < 0.01) promoted reduced ATP production. nMSCs (*p* < 0.001) and sMSCs (*p* < 0.001) were both able to recover mtATP mRNA after OGD, with more robust recovery of ATP production in sMSCs than nMSCs (*p* < 0.001). Additionally, OGD neurons with sMSCs had insignificantly different mtATP mRNA levels compared to the control. These results demonstrate sMSCs’ therapeutic potential to nearly restore ATP production to control levels after OGD in neurons, likely via production of sMito given these variables directly focus on mtROS or mtATP. Overall, these results exemplify the underlying mechanisms of ROS reduction and enhanced ATP production via co-culture with MSCs harnessing healthy mitochondria, which likely contributed to restored viability and metabolism seen in MSC-treated OGD neurons.

## Discussion

Our results showed that MSCs can be primed with metabolic switching to increase basal energy production, spare respiratory capacity (SRC), and overall ATP production, while decreasing proton (H+) leak. Prior research in cancer cells demonstrates that medium switching from glucose to galactose enhances mitochondrial respiration, increases the amount of oxidative phosphorylation components, and increases cristal:inner membrane surface area ratio, suggesting overall enhanced mitochondrial function to combat OGD. In muscle cells, similar enhanced mitochondrial function is observed when cells are cultured in a galactose medium compared to glucose mediums, as measured by oxidative phosphorylation and lactate measurements (Aguer et al., 2011). These relevant glucose–galactose switching protocols in cancer and muscle cells formed the basis of our scientific premise that such paradigm may generate super mitochondria. In our study, such metabolic switching in cultured MSCs similarly generated sMito, which likely contributed to the improved cell viability and cell metabolism of OGD-exposed neurons. The probable mechanisms behind this restored functionality are decreased mtROS and increased mtATP levels.

Mitochondrial damage is known to play a role in cardiac, neurological, and skeletal muscle disease (Johnson et al., 2019). Vital aspects of mitochondrial function include a balanced fusion:fission ratio, minimal H+ leak, adequate ATP balance, and effective SRC. A balance of fusion and fission allow mitochondria to maintain their shape and functionality (Nguyen et al., 2019). While fusion and fission were not measured directly in this study, co-culture of sMSCs restored functionality of OGD-exposed neurons, implying these mitochondria likely produced a cell pro-survival fusion:fission ratio. H+ leak can be used as a measure of mitochondrial damage because the proton gradient should typically be coupled to ATP production (Divakaruni et al., 2014). Accordingly, it is likely beneficial that the co-cultured sMSCs in this study were able to reduce H+ leak and improve mitochondrial efficacy. SRC accounts for the cell’s ability to adapt to increased energy need and how close a cell is working to its maximal respiration (Divakaruni et al., 2014). Insufficient SRC has been shown to promote cell death or senescence (Desler et al., 2012). Restoring SRC may improve stroke outcomes, and our results suggest that sMSCs’ enhanced SRC possibly contributed to neuronal rescue after OGD. ATP production is also vital for mitochondrial function (Dai et al., 2014). Excessive ATP, however, may lead to an inflammatory response or cell death (Idzko et al., 2014; Pontes et al., 2015). The present co-culture of MSCs with the OGD-exposed neurons improved cell viability suggesting that the sMito generated by sMSCs were optimal for cellular repair processes to occur.

The present study reveals mitochondrial rescue-facilitated recovery from ischemic stroke. While our results suggest that the mitochondria might have transferred to the neurons (Hayakawa et al., 2016), future studies are necessary to examine mitochondrial transfer under metabolic switching. Other mechanisms of MSC therapy for OGD propose exosome involvement (Kong et al., 2020); however, our reports of mitochondrial function via increased ATP mRNA, mtROS mRNA, and metabolic activity suggest that sMito generation play a large role in the therapeutic effects of sMSCs for stroke treatment. While mitochondrial damage is only one of the major mechanisms of cell death after stroke, mitochondrial regeneration and transfer may be the basis of a lifesaving new stroke therapy.

## Supplementary Information

Below is the link to the electronic supplementary material.

Supplementary file1 (DOCX 8282 KB)

## Data Availability

All data are available as requested from corresponding author C.V.B.

## Abbreviations

OGD: Oxygen glucose deprivation
MSC: Mesenchymal stem cell
sMito: Super mitochondria
nMito: Normal mitochondria
sMSC: Switched mesenchymal stem cell
nMSC: Normal (control) mesenchymal stem cell
OCR: Oxygen consumption rate
