# Supplementary material for: Metabolic Switching of Cultured Mesenchymal Stem Cells Creates Super Mitochondria in Rescuing Ischemic Neurons
Source: Neuromolecular Med. 2022 Jul 20;25(1):120–4. doi: 10.1007/s12017-022-08720-3 (PMC10025198; doi:10.1007/s12017-022-08720-3)
Supplement: Supplementary file 1 — Supplementary file1 (DOCX 8282 KB) [file 12017_2022_8720_MOESM1_ESM.docx]

**In-Depth Methods**

*MSC Cultures*

Human umbilical cords (hUC; n = 03) were purchased from Zen-Bio and they were obtained after mothers’ informed consent, immediately after full-term births with normal vaginal delivery. The isolation of MSCs from perivascular region of hUC was performed as previously described(Russo et al., 2020). An enzymatic method was chosen for the isolation of MSCs in order to increase the cell harvest yield around the vessels. Briefly, the hUCs were washed in 1x phosphate buffered saline (PBS) in order to remove bloodstains and then rinsed in warm HBSS (Gibco) supplemented by 2x antibiotics/antimycotics (Gibco). Subsequently, the hUC was cut into pieces of about 5–6 cm length and then carefully sectioned longitudinally to expose the blood vessels. For the isolation of MSCs, the blood vessels were isolated using forceps and scalpel and placed in 40 ml of HBSS (Gibco) supplemented with 100 U/mL Type I Collagenase (Sigma) and 0.01 U/mL Hyaluronidase (Stemcell Technologies) in a 50 mL Falcon tube and left to digest in for 4 h at 37 °C. After the digestion was completed, all the vessels were removed from the suspension using forceps. The suspension, containing the cells, was centrifuged at 285 g for 10 min. Subsequently, the supernatant was discarded and the cellular pellet was treated with 50 ml of 0.8% ammonium chloride (Stemcell Technologies) and incubated at room temperature for 5 min to lyse the erythrocytes. Thereafter, the tube was centrifuged for 10 min at 285 g and the supernatant was discarded. The cells, obtained from the perivascular region of each hUC, were counted and plated in one non-coated T-75 tissue culture flask with complete medium and put it in 5% CO_2_ incubator at 37 °C. We achieved confluence of MSCs at around day 14 in vitro, then we either continued the ambient cell culture condition or initiated the metabolic switching paradigm. The latter cell culture condition involved switching glucose (10 mM) with galactose (10 mM) in the media. The alternate switching of glucose (3 days *in vitro*) and galactose (3 days *in vitro*) was performed twice before processing the cells for the Seahorse mitochondrial respiration assay. Additionally, we followed the MSC protocol above for the co-culture with primary rat cortical cells. Briefly, once we reached MSC confluence, we initiated either ambient cell culture condition (nMSCs) or metabolic switching condition (sMSCs), then we co-cultured nMSCs or sMSCs with primary rat cortical cells.

*Mitochondrial respiration assay*

To determine cellular oxygen consumption rate, the Seahorse extracellular flux analyzer XFe96 (102416; Agilent, Santa Clara, CA, USA) was used in combination with sequential injection of various compounds. Oxygen consumption rate measurements were performed following the manufacturer's protocol. On the day of experiments, MSCs were detached from cell culture plates and seeded to a Seahorse 96-well plate (101085–004; Agilent) at 5.0 x 104 cells/well. Briefly, the Seahorse 96-well plate was centrifuged in swing bucket rotator with slow acceleration (4 on a scale of 9) to a max speed of 450 rpm with 0 brake. Then, the plate orientation was reversed and centrifuged again to max speed of 650 rpm with 0 brake. To determine cellular oxygen consumption rate (OCR), the Seahorse extracellular flux analyzer XFe96 (102416; Agilent) was used in combination with sequential injection of various compounds (1 μmol/L oligomycin, 1 μmol/L carbonyl cyanide 4-(trifluoromethoxy) phenylhydrazone (FCCP), 0.5 μmol/L Rotenone and Antimycin A). OCR measurements were performed following the manufacturer's protocol.

### *Rat Primary Cortical Cell Culture*

E18 primary rat cortical cells were used (Neuromics; PC35102). The substrate was coated with 50 μg/ml poly D-lysine (0.15 ml/cm^2^; Sigma [P63407](https://www.ncbi.nlm.nih.gov/protein/P63407)). Cells were diluted with NbActiv1TM (0.2 ml/cm^2^) and grown in coating plates at 37 °C. Half of the cell culture medium was changed every 3 days until the cells were confluent. Once confluence was achieved, OGD was initiated.

*OGD*

Once confluence of primary rat cortical cells was achieved, we initiated the OGD. The cells were initially exposed to Dulbecco's phosphate-buffered saline, then placed in an anaerobic chamber (Plas-Labs, Inc, Lansing MI) containing nitrogen (95%) and carbon dioxide (5%) for 15 min at 37 °C, and finally, the chamber was sealed and incubated for 90 min at 37 °C (hypoxic–ischemic condition). OGD was terminated by changing normal media, and cell cultures reintroduced to the regular CO2 incubator (normoxic condition) at 37 °C for 1 h, which represented a model of “reperfusion”(Kaneko, Tajiri, Shojo, & Borlongan, 2014). After reperfusion, wells were randomly co-cultured with nMSCs or sMSCs, or standard medium, at a concentration of 40,000 cells per well overnight (Supplementary figure 1). This co-culture set-up, the primary rat cortical cells were suspended in the treatment condition using 8-well poly-l-lysine plates, with each treatment condition done in six biological samples. The co-culture was created using a two-chamber system with the primary rat cortical cells in lower chamber and the MSCs in the upper chamber, allowing us to conduct accurate assessments of the primary rat cortical cells without contamination from the MSC population. Cell viability (Trypan blue), metabolic activity (3-[4,5-dimethylthiazol-2-yl]-2,5 diphenyl tetrazolium bromide or MTT), and mRNA levels of mtROS and mtATP were examined in neurons.

*Trypan blue Assay*

Trypan blue (0.2%) exclusion method was conducted and mean viable cell counts were calculated in four randomly selected areas (1 mm^2^, n = 10) to reveal the cell viability after the ischemic‐reperfusion condition. Briefly, within 5 min after adding trypan blue, we digitally captured under microscope (×200) 10 pictures (approximately 100 cells per picture) for each condition, then randomly selected 5 pictures, and counted the number of cells for each individual treatment condition. Normalized cell viability was calculated from the following equation: viable cells (%) = [1.00 − (Number of blue cells / Number of total cells)] × 100.

*MTT Cell Viability Assay*

The colorimetric 3-(4,5-dimethylthiazol-2-yl)-2,5-diphenyltetrazolium bromide or MTT reduction assay was conducted by following the instructions for use of Promega Corporation products (Cell Titer 96, Non-Radioactive Cell Proliferation Assay, Promega Corporation, Madison, WI, USA). This method assessed mitochondrial activity and thus cell viability by measuring the ability of cultured cells to convert yellow MTT to purple formazan dye. The supernatant and the cells were separated from the mixed culture at the end of the 3-h exposure time. Approximately 100 μL DMEM without phenol red was added, then 20 μL of the dye solution was added to each well, and the mixture was incubated on the plate at 37 °C for 3 h in a humidified, 5% CO_2_ atmosphere. After incubation, 100 μL of the solubilization solution/stop mix was added to each well, and the plate was allowed to stand overnight in the humidified, 5% CO_2_ incubator at 37 °C. The absorbance was quantified spectrophotometrically at a wavelength of 570 nm and with a reference wavelength of 900 nm in the BioTek Synergy HT 96-well microplate reader (BioTek Instruments, Inc., Winooski, VT, USA).

*mtROS and mtATP mRNA levels*

At the termination of the OGD, the neurons were processed for detection of mtROS mRNA levels measured using MitoSOX™ Red mitochondrial superoxide indicator (M36008; Invitrogen™, San Diego, CA, USA) and mtATP (adenosine triphosphate) content by performing the Mitochondrial ToxGlo™ assay (G8001; Promega Co., Madison, WI, USA).

### *Statistical analysis*

The data were evaluated using ANOVA followed by post hoc Bonferroni tests. Statistical significance was preset at P < 0.05. Data are presented as mean ± SD.

**
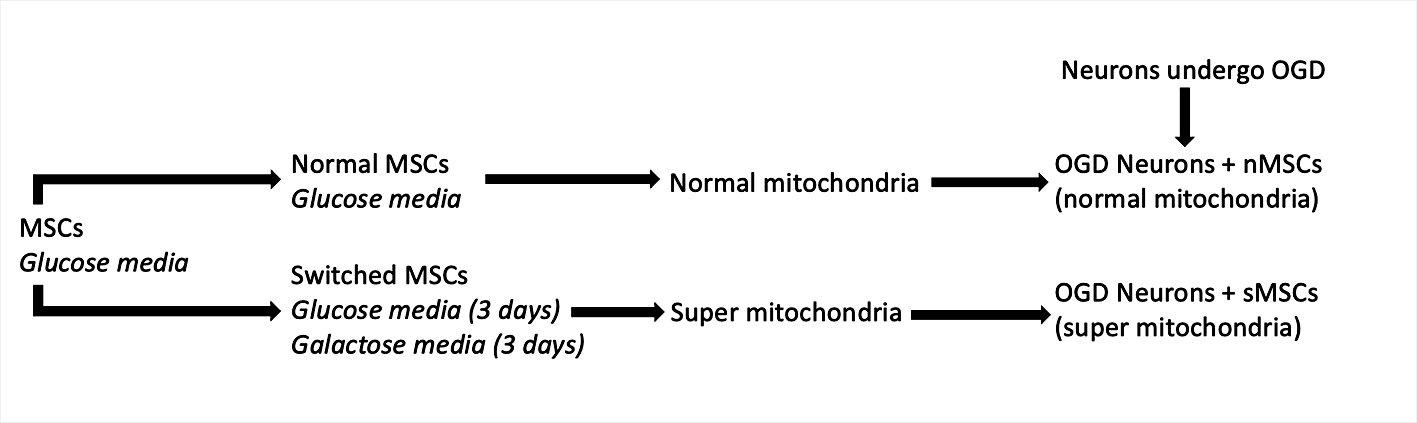
**

**Supplementary Fig. 1** MSC metabolic switching model. Two types of MSCs were created, one using a glucose-supplemented medium (nMSCs), and another with metabolic switching between glucose and galactose medium supplementation every 3 days (sMSCs). The sMSCs generated sMito. Both nMSCs and sMSCs rescued OGD-exposed neurons, but the therapeutic effects were more pronounced in sMSCs.


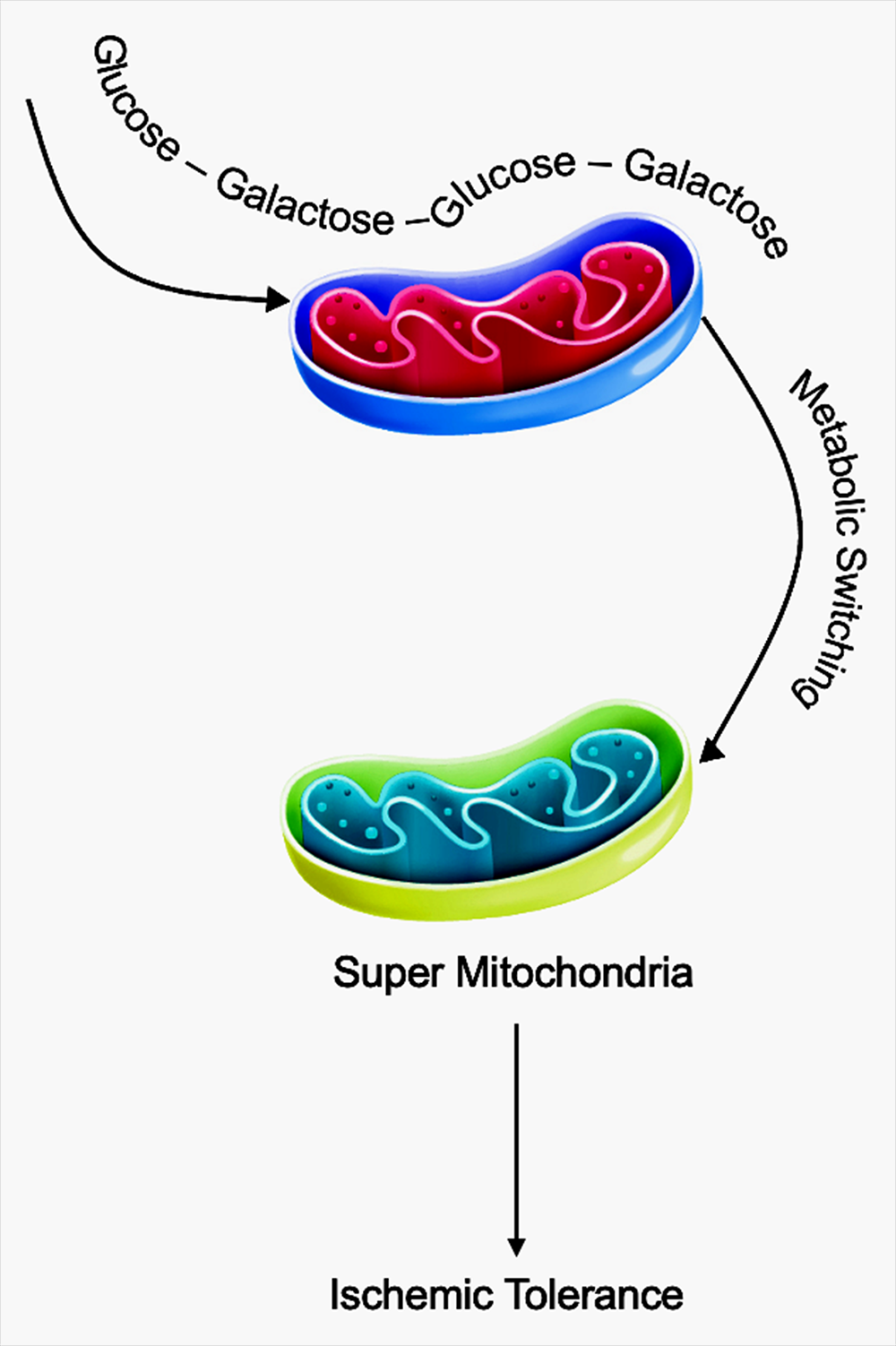


**Supplementary Fig. 2** Metabolic switching and sMito generation. Metabolic switching that involves alternating glucose and galactose in the cell culture medium produces sMSCs with sMito, facilitating ischemic tolerance and affording enhanced rescue when co-cultured with ischemic neurons (Mitochondria vector created by macrovector - www.freepik.com).
